# Regulation of CAR T cell-mediated cytokine release syndrome-like toxicity using low molecular weight adapters

**DOI:** 10.1038/s41467-019-10565-7

**Published:** 2019-06-18

**Authors:** Yong Gu Lee, Haiyan Chu, Yingjuan Lu, Christopher P. Leamon, Madduri Srinivasarao, Karson S. Putt, Philip S. Low

**Affiliations:** 1 Department of Chemistry, Purdue University, West Lafayette, IN 47907 USA; 2 Purdue Institute for Drug Discovery and Purdue Center for Cancer Research, Purdue University, West Lafayette, IN 47907 USA; 3 Endocyte Inc., 3000 Kent Ave, West Lafayette, IN 47906 USA

**Keywords:** Biotechnology, Cancer, Cancer immunotherapy, Immunology

## Abstract

Although chimeric antigen receptor (CAR) T cell therapies have demonstrated considerable success in treating hematologic malignancies, they have simultaneously been plagued by a cytokine release syndrome (CRS) that can harm or even kill the cancer patient. We describe a CAR T cell strategy in which CAR T cell activation and cancer cell killing can be sensitively regulated by adjusting the dose of a low molecular weight adapter that must bridge between the CAR T cell and cancer cell to initiate tumor eradication. By controlling the concentration and dosing schedule of adapter administration, we document two methods that can rapidly terminate (<3 h) a pre-existing CRS-like toxicity and two unrelated methods that can pre-emptively prevent a CRS-like toxicity that would have otherwise occurred. Because all four methods concurrently enhance CAR T cell potency, we conclude that proper use of bispecific adapters could potentially avoid a life-threatening CRS while enhancing CAR T cell tumoricidal activity.

## Abstract (web summary)

Chimeric antigen receptor (CAR) T cell anti-cancer therapies might result in toxic side effects. Here the authors present a strategy based on the modulation of CAR T cells via administration of a bispecific adapter that target them to cancer cells, resulting in diminished CAR-T cells toxicity and enhanced solid tumor eradication.

## Introduction

CAR T cell therapies involve the genetic engineering of a patient’s T cells to express a chimeric antigen T cell receptor (CAR) that can redirect the cell to kill an antigen-expressing cancer cell. In this strategy, a single-chain variable fragment (scFv) that recognizes a tumor antigen is fused to the exoplasmic domain of a T cell receptor to enhance engagement of the T cell with the cancer cell. To ensure rapid killing of the cancer cell, the chimeric antigen receptor is further modified to contain the activation domains of both CD3ζ and another stimulatory receptor (i.e., CD28, 4-1BB, ICOS, OX40, etc.) Equipped with these enhancements, CAR T cells have recently demonstrated the ability to eradicate cancers that have historically been resistant to standard therapies^1,2^. Indeed, treatment of acute lymphoblastic leukemia (ALL) with CAR T cell-based immunotherapies has yielded response rates as high as 70–93% in human clinical trials^3,4^.

While the aforementioned response rates have justifiably generated considerable excitement, use of these genetically engineered T cells to treat human cancers has also introduced challenges associated with control of the CAR T cell’s activity. Thus, concomitant with the augmented cancer cell killing is a heightened release of inflammatory cytokines (i.e., cytokine release syndrome (CRS)) that can in some cases cause life-threating toxicities^5,6^. Fitzgerald et al. have reported that 92% of ALL patients treated with an anti-CD19 CAR T cell therapy experienced a CRS and 50% of these patients developed grade 3–4 symptoms that could have resulted in death^7^.

Although the molecular mechanisms driving a CRS are not fully understood, it has been established that the uncontrolled CAR T cell activation associated with their engagement of tumor cell antigens can induce systemic inflammatory responses similar to those found in hemophagocytic lymphohistiocytosis and macrophage-activation syndrome^8^. As a result, cytokines, such as IFNγ, TNFα, IL-6, GM-CSF, IL-10, IL-2, IL-8, and IL-5 become elevated, leading to pyrexia, hypotension, pulmonary edema, reduced renal perfusion, and various cardiovascular toxicities^9^. To manage these adverse events, tocilizumab (a humanized anti-human IL-6R monoclonal antibody^10^) has often been administered, but in rare cases, even these potent immunosuppressive measures have not prevented CRS-induced death^3,11^. While other antidotes to a severe CRS can involve administration of orthogonal immunosuppressive agents (e.g., corticosteroids^9^) or suicidal elimination of the CAR T cells^12^, these options are less attractive, since they may lead to termination of the therapy.

In an attempt to develop a strategy to sensitively manage a CRS while preserving CAR T cell cytotoxicity, proliferative potential, and survival, we have explored the use of a modified CAR T cell that can only engage a cancer cell when a bispecific adapter is provided to bridge between the CAR T cell and its malignant target. In this strategy, the exoplasmically exposed scFv on the CAR T cell receptor is engineered to recognize fluorescein (i.e., not a tumor antigen), thereby enabling the CAR T cell to kill only cancer cells that have been decorated with fluorescein. Then, to label all cancer cells with fluorescein, a cancer-specific targeting ligand is conjugated to fluorescein, creating a bispecific adapter that binds only cancer cells. In this strategy, tumor cell killing can be controlled by regulating the availability of the bispecific adapter required to bridge between the CAR T cell and its tumor target. Moreover, termination of CAR T cell killing and suppression of any CRS can be achieved by any method that ruptures the bridge tethering the CAR T cell to the cancer cell. In the studies described below, we explore four mechanisms aimed at regulating both formation and rupture of a fluorescein-folate (FITC-folate) bridge that mediates engagement of an anti-fluorescein CAR T cell with a folate receptor (FR)-expressing cancer cell. We demonstrate that both the emergence and intensity of a CRS-like toxicity in mice can be regulated by any method that modulates the number of adapters that form bridges between a CAR T and its cancer cell target.

## Results

### Selection of bispecific CAR T cell-cancer cell adapter

Each of the strategies to regulate a CRS below will be seen to rely on the ability to rapidly change the number of bispecific adapters that mediate engagement of a CAR T cell with its malignant target. Because this number will, in turn, be determined by the rate of entry and clearance of adapters from a tumor mass as well as the “on” and “off” rates of adapter binding to its two receptors, it was important to select a bispecific adapter that would clear with reasonable speed from malignant tissue once its infusion into the cancer patient was terminated. The adapter used in the pilot studies below was FITC-folate, a molecule that in human clinical trials has been shown to have a circulation half-life of <90 min^13^. Based on this short circulation time, we hypothesized that upon interruption of adapter administration, the number of adapter-mediated bridges between a CAR T and cancer cell would fairly rapidly decline, enabling reasonable control over the activation state and cytokine release activity of the CAR T cells. The experiments that follow provide several tests of this hypothesis.

### Characterization of an anti-fluorescein CAR

**Fig. 1:**
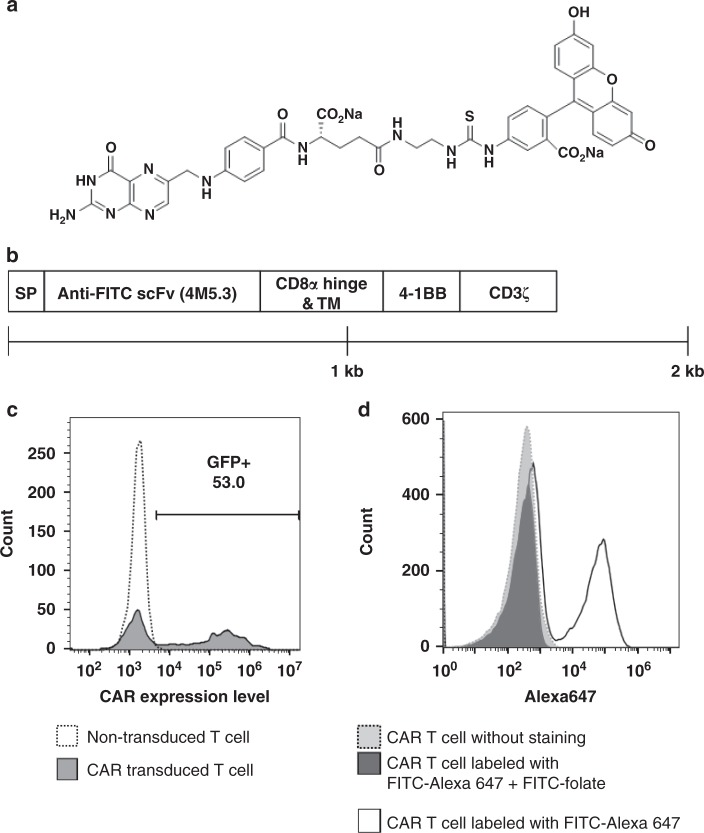
Design and characterization of an anti-fluorescein CAR T cell and fluorescein-folate bispecific adapter. **a** Structure of fluorescein-folate (FITC-folate). **b** Diagram showing the construction of anti-fluorescein CAR, where SP = signal peptide, scFv = single chain variable fragment that recognizes fluorescein with a *K*_D_ = 270 fM, TM = transmembrane domain, 4-1BB = cytoplasmic activation domain from CD137, and CD3ζ = the cytoplasmic activation domain of CD3 zeta. **c** Transduction efficiency of CAR T cells evaluated by flow cytometry. Open histogram: Non-transduced T cells; filled histogram: T cells transduced with lentivirus expressing GFP and the CAR construct shown in (**b**). **d** Demonstration that FITC-folate binds to anti-fluorescein CAR T cell. Filled histogram (gray): anti-fluorescein CAR T cell without staining; open histogram: anti-fluorescein CAR T cell labeled with FITC-Alexa647 (10 nM); filled histogram (black): anti-fluorescein CAR T cell labeled with FITC-Alexa647 (10 nM) in the presence of competing 100-fold excess FITC-folate (1 μM))

Figure 1a shows the structure of our FITC-folate adapter. On the structure’s left resides the vitamin, folic acid, which was selected for tumor targeting because its receptor (FRα) is over-expressed on ~40% of human cancers, but largely absent or inaccessible in normal tissues^14–17^. On the right side lies fluorescein, which was chosen for CAR engagement because a human anti-fluorescein antibody with femtomolar affinity for fluorescein was already described in the literature^18^. Figure 1b summarizes construction of the CAR that was employed to direct the cytotoxic CAR T cells to kill the FRα+ cancer cells. The anti-fluorescein scFv from the aforementioned antibody was fused to CD3 zeta chain of a T cell receptor that was previously engineered to contain the intracellular activation domain of CD137 (4-1BB)^19^. Insertion of this construct into a lentiviral vector allowed transduction of pre-activated human T cells with 50–60% efficiency, as shown by the fraction of T cells staining for GFP (a fluorescent marker co-transduced with the CAR) (Fig. 1c). Figure 1d further demonstrates that the CAR-expressing T cells bind FITC-folate, as established by the ability of FITC-folate to block FITC-Alexa 647 binding to the engineered T cells. The binding affinity of the CAR for fluorescein was measured as *K*_d_ = 30 pM and the affinity of the folate for FRs on MDA-MB-231 cells was determined to be *K*_d_ = 1.0 nM^20^.

**Fig. 2:**
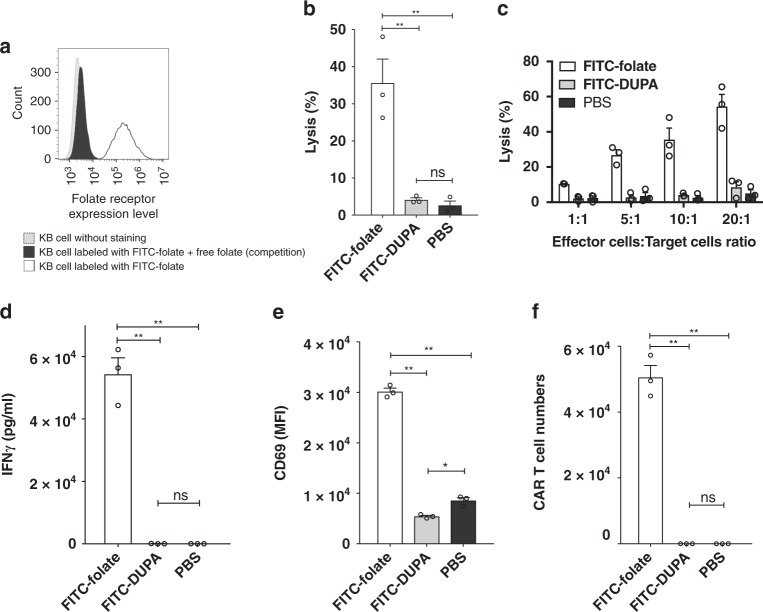
Demonstration that FITC-folate bispecific adapter mediates anti-fluorescein CAR T cell engagement with folate receptor-expressing cancer cells (KB cells). **a** Demonstration that FR is expressed on KB cells. Grey histogram: KB cells without staining; Black histogram: KB cells labeled with 100 nM FITC-folate in the presence of excess (10 μM) free folate as competitor; Open histogram: KB cells labeled with 100 nM FITC-folate. **b** Cytotoxicity of CAR T cells towards KB cells upon addition of correct FITC-folate (100 nM) but not mismatched FITC-DUPA (100 nM) or no (PBS) adapter. **c** Impact of effector:target cell ratio on CAR T cell lysis of KB cells in presence of FITC-folate, FITC-DUPA, or no adapter. **d** IFNγ production is induced by addition of FITC-folate (100 nM) but not FITC-DUPA (100 nM) (after 24 h co-culture). **e** Expression of activation marker (CD69) on anti-fluorescein CAR T cells occurs only upon addition of correct adaptor (after 24 h co-culture). **f** Proliferation of anti-fluorescein CAR T cells is induced by FITC-folate but not FITC-DUPA (after 5 days co-culture). For panels **b**, **d**–**f**, the ratio of anti-fluorescein CAR T cells to KB cells was 10 to 1. Bar graphs represent mean ± s.d. *n* = 3. One-way ANOVA with post-hoc Tukey tests were performed for all comparisons (* denotes a *p-*value < 0.05, ** < 0.01, ns = not significant)

The killing potency of the engineered T cells was first examined by evaluating the ability of FITC-folate adapter to mediate CAR T cell elimination of tumor cells in culture. Figure 2a shows that FITC-folate binds to FR positive KB cells, as demonstrated by (i) the shift in KB cell fluorescence upon addition of FITC-folate, and (ii) the blockade of this shift upon competition with free folic acid. CAR T cell-mediated KB cell killing was then shown by demonstrating lysis of KB cells in the presence (but not absence) of the bridging adapter. Thus, as shown in Fig. 2b, lysis of the cancer cells was observed when both CAR T cells and FITC-folate were present, but not when FITC-folate was absent (PBS) or when FITC-DUPA (an adapter that bridges to prostate cancer cells but not KB cells) was present. Moreover, anti-fluorescein CAR T cells were capable of eradicating KB cells at multiple effector to target cell ratios, but again only when FITC-folate was present (Fig. 2c). Analysis of concurrent production of IFNγ provided further evidence that CAR T cell tumoricidal activity was only triggered by the correct adapter (Fig. 2d), and related experiments established that both anti-fluorescein CAR T cell proliferation and CAR T cell activation also required addition of the correct bispecific adapter (Fig. 2e, f). Indeed, expansion of the same CAR T cells in vivo was found to require both the presence of FR-expressing solid tumor and the receptor-matched bispecific adapter. Taken together, these data demonstrate that anti-fluorescein CAR T cell functionality is dependent on the availability of the correct tumor-specific adapter to mediate engagement of the CAR T cell with the desired cancer cell.

### Establishment of cytokine-induced toxicity

**Fig. 3:**
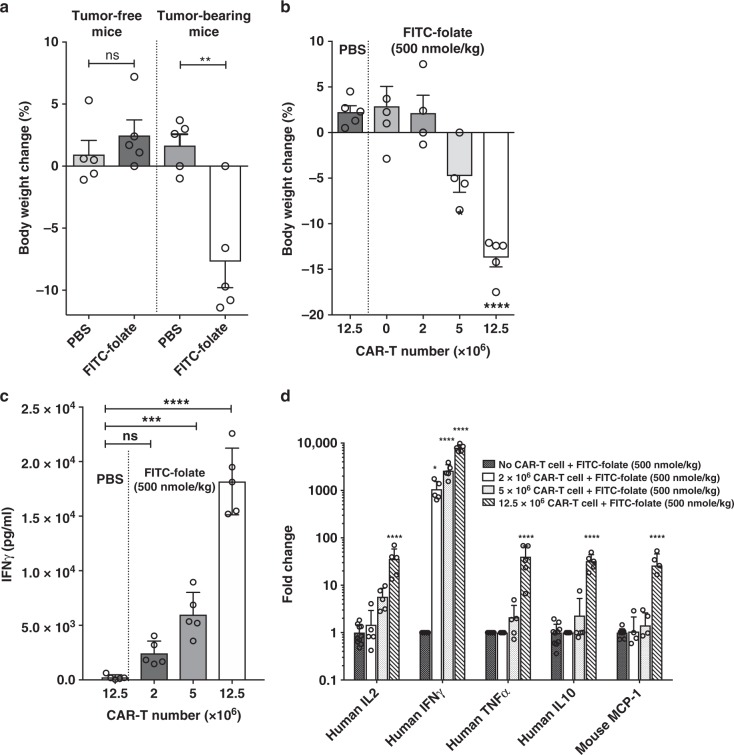
Dependence of CRS on the presence of anti-fluorescein CAR T cells, folate receptor-positive cancer cells (MDA-MB-231 cells) and FITC-folate adapter in vivo. **a** Bodyweight change (%) determined 4 days after anti-fluorescein CAR T cell (5 × 10^6^) infusion into either tumor-free or tumor-bearing mice in the presence or absence of FITC-folate (500 nmole/kg administered on days 1 and 2). **b** Effect of CAR T cell number on bodyweight change (%) (day 4) in tumor-bearing mice after administration of either PBS or 500 nmole/kg FITC-folate on days 1 and 2. **c** Effect of CAR T cell number in tumor-bearing mice on plasma IFNγ concentration on day 4. **d** Effect of CAR T cell number (injected on day 1) on serum cytokine concentrations (determined on day 4) following administration of 500 nmole/kg FITC-folate on days 1 and 2. *n* = 5 mice per group. Bar graphs represent mean ± s.e.m. A *t*-test for panel **a** and one-way ANOVA with post-hoc Dunnett tests were performed for all other panels (*denotes a *p-*value < 0.05, ** < 0.01, *** < 0.001, **** < 0.0001, ns = not significant)

To explore whether manipulation of the duration, concentration or frequency of bispecific adapter dosing might allow control of a CRS, it was necessary to identify conditions where a prominent CRS could be reproducibly induced. Based on protocols found by others to promote a CRS-like toxicity in different immunodeficient mouse strains^21–25^, we undertook to optimize conditions where NSG mice implanted with FR positive MDA-MB-231 cells would display CRS-like symptoms each time they were treated with anti-fluorescein CAR T cells plus FITC-folate. As shown in Fig. 3a, bodyweight loss of ~8% was observed in tumor-bearing mice within 4 days of injection of 5 × 10^6^ CAR T cells plus 500 nmoles/kg FITC-folate; i.e., suggesting the possible occurrence of a CRS^26^. In contrast, no weight loss was observed in tumor-bearing mice injected with CAR T cells in the absence of FITC-folate. Moreover, no weight loss was again detected in tumor-free mice treated with the same anti-fluorescein CAR T cells, regardless of whether adapter was present or not. These data demonstrate that even in the presence of both CAR T cells and FITC-folate, CAR T cell-induced bodyweight loss does not occur unless a fluorescein-decorated cancer cell can be engaged by a CAR T cell. Because FR expression in healthy tissues is primarily restricted to the apical surface of the proximal tubule cells of the kidneys (where FR are inaccessible to immune cells^27–29^), little or no CAR T cell activation was expected in tumor-free mice. Panel A shows that this expectation was realized. Based on these data and additional observations that will be presented below, we believe that bodyweight loss constitutes a reliable measure of cytokine-induced toxicity (i.e. CRS, see also ref. ^19^).

To further establish that the intensity of a cytokine-induced toxicity depends on the number of CAR T cells that form immunological synapses with cancer cells, the dependence of bodyweight loss and IFNγ release was determined as a function of CAR T cell numbers. As shown in Fig. 3b, administration of adapter alone promoted no bodyweight loss. Similarly, injection of 12.5 × 10^6^ CAR T cells alone stimulated no decrease in bodyweight; i.e. confirming that engagement of a cancer cell by the CAR T cell is required for systemic cytotoxicity. While injection of 2 × 10^6^ CAR T cells plus 500 nmole/kg adapter also induced no apparent weight loss, infusion of 5 × 10^6^ or 12.5 × 10^6^ CAR T cells plus the same adapter dose promoted increasingly greater decreases in bodyweight. That these decrements in body mass arose from cytokine-induced toxicity is suggested by concurrent analyses of IFNγ release, which displayed a similar dependence on CAR T cell numbers and the presence of adapter (Fig. 3c). Indeed, quantitative analysis of the rise in human (i.e., CAR T cell derived) IL-2, IL-10, IFN-γ, and TNF-α concentrations upon elevation of injected CAR T cells revealed that the above cytokines can increase by an average of 30- to 8000-fold when tumor-bearing mice that have been infused with 12.5 million CAR T cells are subsequently treated with FITC-folate adapter. And consistent with the dramatic activation of the injected human CAR T cells, a 30-fold upsurge in the murine macrophage/monocyte-derived murine MCP-1 was also observed in the same mice. (Fig. 3d). The fact that the level of increase in both CAR T cell- and host-derived cytokines correlates with the amount of animal weight loss (Supplemental Fig. 1) further confirms that the rise in toxic cytokine levels contributes to bodyweight loss. Collectively, these data demonstrate that the inflammatory cytokines released from both infused human CAR T cells and endogenous murine immune cells can induce CRS-like symptoms in NSG mice once the bispecific adapter is injected to mediate engagement of the CAR T cells with the implanted cancer cells.

### Termination of a pre-existing cytokine-induced toxicity

**Fig. 4:**
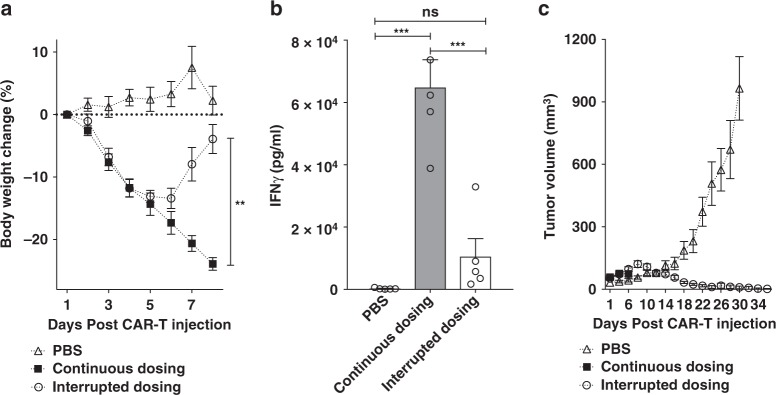
Control of CRS intensity by interruption of bispecific adapter administration. **a** Analysis of bodyweight change (%) as a measure of CRS intensity after administration of a high dose of anti-fluorescein CAR T cells (15 × 10^6^) in either absence (PBS) or presence of FITC-folate (500 nmole/kg administered on days 1, and 2, and alternate days thereafter). In the interrupted dosing regimen, the continuous dosing schedule was followed except FITC-folate injections were omitted on days 4 and 6. **b** Analysis of IFNγ levels in mouse plasma on day 6 using the dosing regimens described in **a**. **c** Measurement of tumor volumes in mice treated as described in **a**. *n* = 5 mice per group. Data represent mean ± s.e.m. One-way ANOVA with post-hoc Tukey tests were performed for all comparisons (*denotes a *p-*value < 0.05, ** < 0.01, *** < 0.001, ns = not significant)

With conditions for inducing a cytokine-induced toxicity in tumor-bearing NSG mice established, the question next arose whether the cytokine-induced toxicity might be suppressed by modulating the level of engagement of the CAR T cells with their cancer targets. To explore this possibility, we first initiated a cytokine-induced toxicity as outlined above and then examined whether interruption of FITC-folate administration might promote cessation of the cytokine-induced toxicity. Figure 4a shows that a single treatment of tumor-bearing mice with 15 × 10^6^ anti-fluorescein CAR T cells followed by alternate-day dosing of 500 nmole/kg FITC-folate promoted rapid and continuous weight loss, leading to the required euthanasia of the five mice by day 8. In contrast, identically treated mice in which FITC-folate dosing on days 4 and 6 was simply omitted (but which received continuous alternate day dosing thereafter) showed only a moderate weight loss from which they rapidly recovered. That this weight loss was related to cytokine production is demonstrated by the related changes in IFNγ levels measured in each mouse’s plasma (Fig. 4b). More importantly, temporary interruption of alternate day dosing not only failed to compromise anti-tumor activity, but instead actually enhanced CAR T cell potency. As shown in Fig. 4c, whereas untreated mice displayed a rapid increase in tumor growth, mice exposed to interrupted adapter dosing experienced complete remission of their tumors. These data suggest that a pre-established cytokine-induced toxicity can be controlled by temporary discontinuation of adapter dosing without subverting CAR T cell cytotoxicity.

**Fig. 5:**
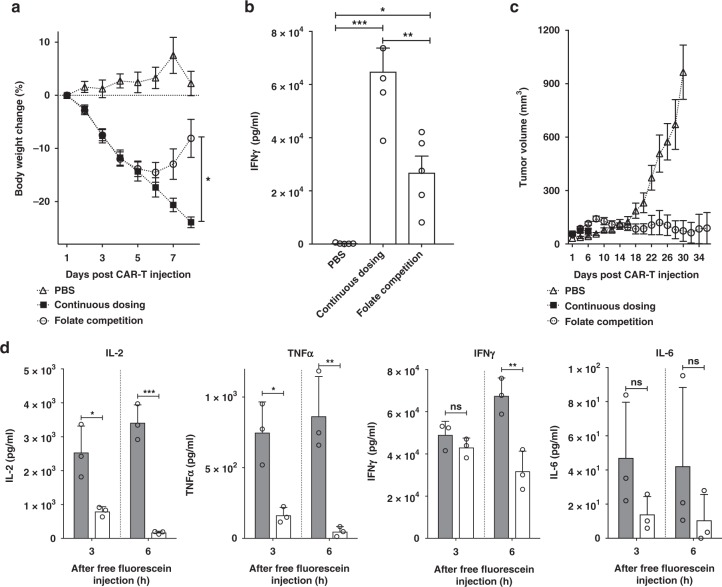
Effect of blockade of adapter binding via competition with free folate or free fluorescein on CAR T cell-mediated cytotoxicity. **a** Measurement of bodyweight change (%) after administration of anti-fluorescein CAR T cells (15 × 10^6^) in the absence (PBS) or presence of FITC-folate (500 nmole/kg administered on days 1, and 2, and alternate days thereafter). For competition studies, 100-fold excess folate was co-injected on days 4 and 6. **b** Analysis on day 6 of IFNγ levels in plasma of above treatment groups. **P < 0.005. **c** Measurement of tumor volume in same treatment groups. *n* = 5 mice per group. Error bars represent mean ± s.e.m. **d** Analysis of time dependence of cytokine levels in plasma of CAR T cell after administration of 12-fold excess free fluorescein to suppress CRS. CRS was induced in all mice by injection of 10 × 10^6^ anti-fluorescein CAR T cells plus FITC-folate (500 nmole/kg on day 3). On day 4, to suppress a potent CRS, 6 μmole/kg free fluorescein was injected, and the indicated cytokines were measured in plasma at 3 and 6 h after administration of fluorescein. *n* = 3 mice/group. Filled bar (gray): no free fluorescein, open bar: free fluorescein. Data represent mean ± s.d. One-way ANOVA with post-hoc Tukey tests were performed for **a** and **b** while a *t*-test was used for comparisons in **d** (* denotes a *p-value* < 0.05, ** < 0.01, *** < 0.001, ns = not significant)

Realizing that transient interruption of adapter dosing could decrease cytokine-induced toxicity, we next wondered whether a more potent decrement in cytokine-induced toxicity might be promoted by the addition of ligands that competed with FITC-folate bridging of CAR T cells to cancer cells. For this purpose, we initiated a cytokine-induced toxicity as described above, and while continuing an uninterrupted alternate day adapter dosing we simply administered 100-fold excess free folate on days 4 and 6 to block adapter bridging. As shown in Fig. 5a, injection of excess folic acid rapidly reversed the weight loss observed with the uninterrupted adapter dosing. Moreover, as seen in Fig. 4, this competition with adaptor not only reduced IFNγ levels in the treated mice (Fig. 5b), but also enhanced CAR T cell potency (Fig. 5c). These data suggest that transient administration of a benign competing ligand (i.e. a vitamin) can terminate cytokine-induced toxicity without compromising tumor therapy.

While folic acid can be used to control cytokine-induced toxicity whenever FITC-folate is used to bridge between a CAR T and cancer cell, it seemed prudent to also examine the ability of fluorescein to block cytokine-induced toxicity, since fluorescein should also compete for FITC-ligand binding regardless of the nature of the tumor-specific ligand in the bispecific adapter. Therefore, cytokine-induced toxicity was induced as described above, and its discontinuation was attempted by administration of competing fluorescein on day 3 of the CRS. As seen with folate, injection of fluorescein caused both a rapid decrease in cytokine-induced toxicity and an increase in therapeutic efficacy. Thus, all CRS-relevant cytokines declined within 3 h of fluorescein administration, with IL-2, TNF-α, and IL-6 decreasing >50% over this short timespan (Fig. 5d). Although IFNγ decreased more slowly, it still demonstrated a decline by 6 h post fluorescein injection, suggesting its production is also suppressed by fluorescein addition. Importantly, plasma concentrations of IL-2, TNF-α, and IL-6 had all diminished to nearly background levels by 6 h; i.e., confirming that a cytokine-induced toxicity can be rapidly controlled by competitive inhibition of adapter bridging.

### Prevention of occurrence of cytokine-induced toxicity

**Fig. 6:**
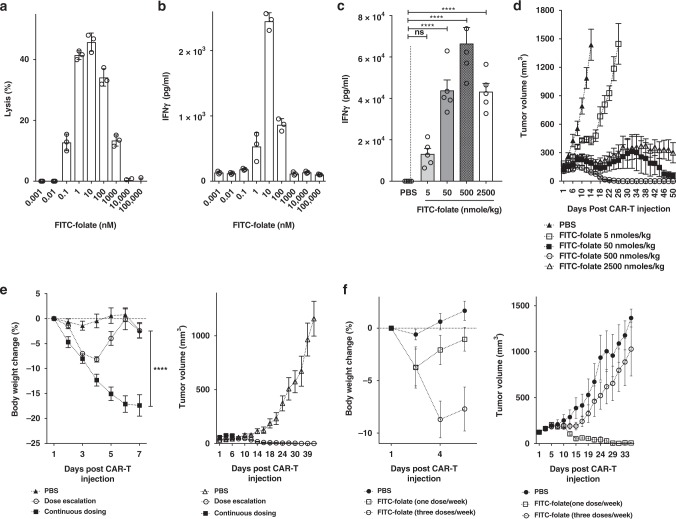
Prevention of CRS by gradual escalation of adapter dose or decrease in adapter dosing frequency. **a**–**d** Effect of adapter concentration on the regulation of cytokine release and anti-tumor activity in vitro and in vivo. **a** Lysis of MDA-MB-231 cells in culture by anti-fluorescein CAR T cells (5:1 = effector:tumor cell ratio) in the presence of various concentrations of FITC-folate (0.001 nM to 100 μM). **b** IFNγ release from cells described in (**a**). **c** The level of IFNγ in the plasma of MDA-MB-231 tumor-bearing mice 6 days after initiation of therapy by injection of 15 × 10^6^ CAR T cells. Mice were treated with 5, 50, 500, or 2500 nmole/kg FITC-folate on days 1, 2, 8, 10 and alternate days thereafter (treatments on days 4 and 6 were omitted to avoid a CRS). **d** Analysis of tumor growth in treatment groups of panel **c**. **e** Measurement of body weight change and tumor volume in MDA-MB-231 tumor-bearing mice treated with 15 × 10^6^ CAR T cells plus (1) PBS only, (2) gradually escalating dose of FITC-folate (0.5 nmole/kg on days 1 and 2, 5 nmole/kg on days 4 and 6, 50 nmole/kg on days 8 and 10, and 500 nmole/kg from day 12 onward), or (3) constant dose of 500 nmole/kg FITC-folate on alternate days. **f** Measurement of body weight change and tumor volume in MDA-MB-231 tumor-bearing mice treated with 5 × 10^6^ CAR T cells on day 1 plus either PBS or FITC-folate (500 nmole/kg) on day 1, or alternatively on days 1, 3, and 5. *n* = 5 mice per group. All data represent mean ± s.e.m. One-way ANOVA with either post-hoc Dunnett (**c**) or Tukey tests (**e**) were performed (*denotes a *p*-value < 0.05, ** < 0.01, *** < 0.001, **** < 0.0001, ns = not significant)

Although a highly elevated and prolonged CRS can lead to patient death^7,30^, some level of CRS in ALL is typically indicative of CAR T cell engraftment and effector cell activation whereas the absence of CRS is associated with engraftment failure (Michael Jensen, personal communication). The question, therefore, arose whether optimization of either adapter or CAR T cell dose could prevent CRS without loss of anti-tumor activity. To explore this possibility, we first examined the effect of adapter dose on tumor cell lysis and IFNγ release in vitro. For this purpose, anti-fluorescein CAR T cells were added to MDA-MB-231 cultures and treated with increasing FITC-folate concentrations from 0.001 to 100,000 nM. As shown in Fig. 6, adapter doses below 0.01 nM exerted little effect on tumor cell lysis and only minimal effect on IFNγ production. In contrast, somewhat higher concentrations wielded a more potent impact on both parameters, whereas still higher doses promoted a reduction in both cytokine release and MDA-MB-231 cell killing. Based on the mechanism of adapter bridging, it was predicted that excess adapter would saturate all sites on both CAR T cells and cancer cells, thereby blocking normal bivalent bridging between the two cell types; i.e. resulting in a decrease in CAR T cell activity.

To evaluate the same adapter concentration dependence in vivo, MDA-MB-231 cells were implanted in NSG mice and tumor growth and cytokine release were again monitored as a function of FITC-folate dose. Unfortunately, as seen in Fig. 4, continuous alternate day dosing resulted in the anticipated severe cytokine-induced toxicity, regardless of adapter dose, forcing us to omit bispecific adapter administration on days 4 and 6. However, even with this modification, analysis of the effect of adapter concentration was possible, revealing a bell-shaped dependence similar to that observed in vitro; i.e., an initial increase followed by a decrease in IFNγ concentration as adapter dose was elevated (Fig. 6c). Importantly, tumor shrinkage also displayed a bell-shaped dose-response, with 500 nmole/kg exhibiting complete tumor eradication, but lower and higher concentrations displaying lower potencies (Fig. 6d). These observations confirm that maximal bridging between CAR T and cancer cells only occurs at intermediate adapter concentrations, where sufficient adapter is present to maximize bridge formation, but excess adapter has not yet resulted in a competition for the bridging function.

With the ability to exploit adapter dose to regulate CAR T cell activation established, we next investigated whether an adapter dosing regimen might be identified that could prevent emergence of a severe cytokine-induced toxicity without compromising anti-tumor potency. Based on the hypothesis that cytokine-induced toxicity becomes most severe when CAR T cells are activated too precipitously, we elected to test two less aggressive adapter dosing schedules that might more gradually induce CAR T cell activation. First, FITC-folate concentration was increased gradually from 0.5 to 500 nmole/kg during each successive administration of the adapter. As shown in Fig. 6e, this slow escalation of adapter dose caused only a transient weight loss that resolved without intervention. In contrast, continuous dosing of 500 nmole/kg caused a dramatic and continuous weight loss that forced eventual euthanasia of the mice. More importantly, gradual dose escalation also resulted in complete cures of the mice, whereas constant dosing provided little therapeutic benefit (Fig. 6e). These data argue again that anti-tumor activity can be uncoupled from CRS and that slower CAR T cell activation may, in fact, enhance tumoricidal activity while reducing CRS.

Finally, we also examined the impact of reducing adapter dosing frequency in the hope that longer intervals between CAR T cell activation might permit partial CAR T cell relaxation and thereby reduce any exhaustion that might normally arise from continuous and chronic exposure to antigen^31,32^. As shown in Fig. 6f, while 3 doses/week of bispecific adapter caused significant weight loss, a single FITC-folate injection per week caused no weight loss. Moreover, as seen in many of our previous studies, anti-tumor activity was observed to increase as cytokine-induced toxicity intensity appeared to decrease; i.e. yielding complete cures when only one dose of adapter was administered per week.

## Discussion

Persistence of a severe CRS has been associated with deaths during CAR T cell clinical trials^30,33^. According to literature reports, these deaths may have derived from sustained exposure to cytokines that are produced during uncontrolled activation of the CAR T cells. Upon encountering a tumor antigen, CAR T cells are engineered to both kill the engaged tumor cell and proliferate (both mediated in part by release of cytokines), leading to a positive feedback-mediated expansion in CAR T cell numbers. Because this growth in the CAR T cell population enables a further increases in tumor cell engagement, the natural consequence is a spiraling increase in cytokine levels that can eventually become toxic.

Current methods to control CRS involve either: (i) neutralization of inflammatory cytokines^3,11^, (ii) administration of anti-inflammatory drugs^9^, or (iii) activation of engineered suicide genes that induce CAR T cell death^12^. Because all three methods require suppression of CAR T cell activity, oncologists must often decide between suppressing a CRS and inhibiting its therapeutic potency. As noted above, use of a low molecular weight bispecific adapter to control engagement of CAR T cells with cancer cells can permit manipulation of CAR T cell activation without loss of tumoricidal activity. Thus, as shown in Figs. 4 and 5, a pre-existing cytokine-induced toxicity can be readily controlled without compromising anti-tumor potency by either (i) omitting two consecutive doses of adapter, or (ii) disrupting adapter bridging via addition of a competing ligand. Because both folate and fluorescein are FDA-approved, use of either competitor to block adapter bridging should constitute a nontoxic strategy to reduce a CRS.

In an effort to pre-emptively prevent a cytokine-induced toxicity before its inception, we also explored different adapter dosing regimens that might lead to more gradual CAR T cell activation. In this case, avoidance of cytokine-induced toxicity induction was achieved by either (i) gradually escalating adapter dose, or (ii) decreasing the frequency of adapter administration, again without loss of tumoricidal activity. The mechanism by which continuous dosing at 500 nmol/kg adapter invariably induces a cytokine-induced toxicity, whereas gradual dose escalation to 500 nmol/kg followed by continuous dosing at this same concentration avoids a cytokine-induced toxicity was never resolved. However, it was repeatedly observed that whenever this cytokine-induced toxicity was averted, the potency of the therapy was concurrently improved. Although the factors underpinning this relationship were also never elucidated, one hypothesis that perhaps warrants further scrutiny is that sustained activation of the CAR T cells can lead to their exhaustion, and that a gradual increase in antigen exposure or a dosing schedule that includes regular episodes of reduced antigen stimulation can similarly avoid driving the CAR T cells into this unresponsive state.

Other groups have also employed bispecific adapters to mediate CAR T cell engagement with tumor cells, only in all of these cases high molecular weight antibodies/antibody fragments have been used as the cancer-binding components of their bispecific adapters^34–39^. Although excellent anti-tumor activity has often observed with these macromolecular adapters, no studies aimed at controlling a CAR T cell derived toxicity were ever reported except a comment by Ma et al.^36^ that administration of an initial low dose of FITC-anti-CD19 adapter followed by a higher dose of the same adapter caused less toxicity than two consecutive administrations at the higher adapter dose. While administration of such protein adapters can admittedly promote potent engagement of an engineered CAR T cell with its cancer cell target, the flexibility and speed associated with control of any emerging CRS will almost certainly be less than that seen with much smaller adapter molecules. Thus, most antibodies (MW~150,000) have circulation half-lives of several days to weeks in humans^40–42^, preventing rapid manipulation of their concentrations and thereby delaying any response to a change in their dosing schedules. In contrast, FITC-folate (MW~873) has a circulation half-life of <90 min in humans^13^, allowing for facile management of its concentration by either discontinuing its administration or injecting a competing ligand. As a consequence, simple omission of an adapter dose (e.g., Fig. 4) or addition of free folate or fluorescein (e.g., Figs. 4 and 5) was found to induce a rapid change in CRS intensity. In fact, as shown in Fig. 5, one injection of sodium fluorescein (an FDA approved drug^43,44^) promoted a > 50% reduction in cytokine levels in <3 h. Assuming that somewhat similar kinetics can be achieved in humans, the danger of a lethal CRS can hopefully be reduced.

## Methods

### Cell lines and T cells

FR positive cell lines (e.g., KB and MDA-MB-231) were maintained in folic acid free RPMI 1640 (Gibco, Ireland) containing 10% heat-inactivated fetal calf serum and 1% penicillin–streptomycin in 5% CO_2_ at 37 °C. KB and MDA-MB-231 obtained from American Type Culture Collection (ATCC). According to ATCC, KB cells may be contaminated with HeLa cells and authentication of KB and MDA-MB-231 were carried out by short-tandem repeat (STR) analysis. Cell lines were not tested for mycoplasma contamination. Peripheral blood mononuclear cells (PBMC) were isolated by Ficoll density gradient centrifugation (GE Healthcare Lifesciences, USA) of fresh human blood from healthy volunteers (IRB#: 1702018875). Pure CD3^+^ T cells were isolated from PBMCs using EasySep™ Human T Cell Isolation Kit (STEM CELL technologies, Canada) and then cultured in TexMACS^TM^ medium (Miltenyi Biotech Inc, CA) containing 1% penicillin and streptomycin sulfate in the presence of human IL-2 (100 IU/ml, Miltenyi Biotech Inc, CA). T cells were divided and the above media was changed every 2–3 days.

### Generation of lentiviral vector with anti-fluorescein CAR

An overlapping PCR method was used to generate the CAR construct containing a scFv against fluorescein. The coding sequence for the scFv was synthesized (GeneScript, NJ) from an affinity optimized sequence of a human anti-fluorescein antibody, 4M5.3 (Kd = 270 fM, 762 bp)^18^. Sequences encoding the human CD8α signal peptide (SP, 63 bp), the hinge and transmembrane regions of CD8α (249 bp), the cytoplasmic domain of 4-1BB (CD137, 141 bp) and the cytoplasmic domain of CD3ζ chain (336 bp) (purchased from GeneScript) were fused with the anti-fluorescein scFv by overlapping PCR. The resulting CAR construct (1551 bp) was inserted into EcoRI/NotI cleaved lentiviral expression vector pCDH-EF1-MCS-(PGK-GFP) (System Biosciences, CA) and expanded/purified using PureLink Hipure plasmid midiprep kit (Invitrogen, CA). The sequence of the CAR construct in lentiviral vector was confirmed by DNA sequencing (Purdue Genomic Core Facility, IN).

### Production of lentivirus and human T cell transduction

To prepare lentivirus containing the anti-fluorescein (scFv) CAR, 293TN packaging cell line was cotransfected with lentiviral vector encoding anti-fluorescein scFv CAR and a 2nd generation mixture of packaging plasmids (Cellecta, CA). After 24 and 48 h transfection, supernatants containing lentivirus encoding the CAR gene were harvested and virus particles were concentrated using a standard polyethylene glycol virus concentration method (Clontech, CA).

### Transduction of human T cell with CAR-expressing lentivirus

Isolated T cells (see above) were activated using Dynabeads coupled to anti-CD3/CD28 antibodies (Life Technologies, CA) for 12–24 h in the presence of human IL-2 (100 IU/ml) and then transduced with the aforementioned lentivirus encoding both GFP and the anti-fluorescein CAR. After 72 h transduction, T cells were analyzed for GFP fluorescence by flow cytometry to determine transduction efficiency.

### Synthesis of fluorescein-folate and fluorescein-PSMA

FITC-folate was synthesized by coupling FITC (1.1 equiv) with folate-ethylenediamine (1.0 equiv) in anhydrous dimethylsulfoxide. The crude product of this reaction was precipitated with ether. After drying under vacuum, the resulting product was purified by high-performance liquid chromatography (HPLC) and analyzed by mass spectrometry.

FITC−DUPA was synthesized by solid phase methodology using a Universal NovaTag resin. Fifty mg of resin was swollen with dichloromethane (DCM) and dimethylformamide (DMF) and the fmoc group was then deprotected with a solution of 20% piperidine in DMF. The resin was then treated with a solution of DUPA(OtBu)-OH (1.5 equiv), 1-[bis(dimethylamino)methylene]-1H-1,2,3-triazolo[4,5-b]pyridinium 3-oxid hexafluorophosphate (HATU, 2.5 equiv) and diisopropylethylamine (DIPEA, 4.0 equiv) in DMF and after the completion of reaction, washed with DMF, DCM and isopropanol. After swelling the resin in DCM, the resin was reacted with a solution of 1 M N-hydroxybenzotriazole in DCM/trifluoroethane (TFE) (1:1). After swelling the resin in DMF, the resin was reacted with a solution of Fmoc-Phe-OH (2.5 equiv), HATU (2.5 equiv) and DIPEA (4.0 equiv) in DMF. This coupling and deprotecting sequence was repeated for 2 more coupling steps for 8-aminooctanoic acid and fluorescein isothiocyanate. At the end, FITC−DUPA was cleaved from the resin using a trifluoroacetic acid (TFA):H2O:triisopropylsilane:cocktail (95:2.5:2.5) and concentrated under vacuum. The concentrated product was precipitated in diethyl ether and dried. The resulting product was purified using HPLC and analyzed by mass spectrometry.

### Binding of bispecific adapter to target receptors

To examine the ability of these bispecific adapters to bind anti-fluorescein scFv on CAR T cells, a competitive binding assay had to be developed, because measurement of the fluorescein signal from CAR T cell bound bispecific adapter could not be used due to the overlap of its fluorescence with that of the GFP expressing CAR T cells. For this purpose, FITC-Alexa647 (10 nM) was allowed to bind anti-fluorescein CAR T cells in the absence or presence of excess (1 μM) competing ligand (i.e., FITC-folate) for 1 h at room temperature. After incubation, anti-fluorescein CAR T cells were washed 3x with PBS to remove unbounded FITC-Alexa647, and the washed cells were analyzed for Alexa647 fluorescence by flow cytometry. For analysis of FITC-folate to binding to FR, FR positive KB cells were incubated with FITC-folate (100 nM) in the absence or presence of excess (10 μM) free folate (i.e., as a competitive ligand). The cells were washed 3× with PBS and analyzed by flow cytometry for FITC-folate binding.

### Anti-tumor activity of anti-fluorescein CAR T cells in vitro

FR positive cancer cell lines (e.g., KB or MDA-MB-231 cells) were seeded at density of 10^4^ cells/100 μl media into 96 well plates and grown overnight. Anti-fluorescein CAR T cells were added to each well in the absence or presence of bispecific adapters. After co-incubation for 6–24 h, plates were centrifuged at 350xg for 10 min to remove debris and supernatants were analyzed for lactate dehydrogenase release (cell death analysis) using Pierce^TM^ LDH cytotoxicity assay kit (Thermo Fisher Scientific, MA) and interferon γ (IFNγ) levels using human IFNγ ELISA kit (Biolegend, CA), while pellets were either evaluated for CAR T cell activation by staining with anti-human CD69 APC (1:100 dilution, Biolegend, catalog#: 310910) or examined for CAR T cell proliferation by culturing for 5 days in TexMACS^TM^ medium (Miltenyi Biotech Inc, CA) containing 1% penicillin and streptomycin sulfate and quantitating by flow cytometry using the intrinsic GFP fluorescence and staining with anti-human CD3 APC antibody (1:100 dilution, Biolegend, catalog#: 300312).

### Anti-tumor activity of anti-fluorescein CAR T cells in vivo

Immunodeficient NSG mice (Jackson Laboratory, ME) were implanted subcutaneously with MDA-MB-231 cells and injected intravenously with CAR T cells and then FITC-folate (as indicated) when tumors reached ~100 mm^3^ in size. Mice were maintained on folic acid-deficient diet (TD.95247, Envigo) in order to reduce the level of folic acid in mice to a physiological levels found in humans^45,46^. Tumors were measured every other day with calipers, and tumor volume was calculated according to equation: Tumor volume = ½(L × W^2^) where L is the longest axis of the tumor and W is the axis perpendicular to L. Mouse blood was also collected to measure cytokine levels (e.g., IL-2, IL-6, IFNγ, and TNFα) using LEGENDplex bead-based immunoassay (Biolegend, CA) and systemic toxicity was monitored by measuring bodyweight loss. All animal care and use followed by National Institutes of Health (NIH) guidelines and all experimental protocols were approved by the Purdue Animal Care and Use Committee.

### Statistical analyses

GraphPad Prism version 7 software (Graphpad; San Diego, CA) was used for statistical analyses. All figures reported mean ± s.e.m values unless otherwise noted. For comparison of two groups, a 2-tailed *t*-test was utilized while multiple groups were compared using a 1-way ANOVA followed by either a Tukey or Dunnett post hoc analysis where appropriate.

## Supplementary information

Supplementary Information

## Data Availability

The data that support the findings of this study are available within the article and its supplementary files or from the corresponding author upon reasonable request.
