# Supplementary material for: Regulation of CAR T cell-mediated cytokine release syndrome-like toxicity using low molecular weight adapters
Source: Nat Commun. 2019 Jun 18;10:2681. doi: 10.1038/s41467-019-10565-7 (PMC6581910; doi:10.1038/s41467-019-10565-7)
Supplement: Supplementary file 1 — Supplementary Information [file 41467_2019_10565_MOESM1_ESM.pdf]

Regulation of CAR T Cell-mediated Cytokine Release Syndrome-like Toxicity using Low Molecular Weight Adapters

Yong Gu Lee[1], Haiyan Chu[2], Yingjuan Lu[2], Christopher P. Leamon[2], Madduri Srinivasarao[1], Karson S. Putt[1] and Philip S. Low[1*]

1. Department of Chemistry, Purdue Institute for Drug Discovery, and Purdue Center for Cancer Research, Purdue University, West Lafayette IN 47907
2. Endocyte Inc., 3000 Kent Ave, West Lafayette IN 47906

*Corresponding author: Philip S. Low (plow@purdue.edu)

Supplemental figures

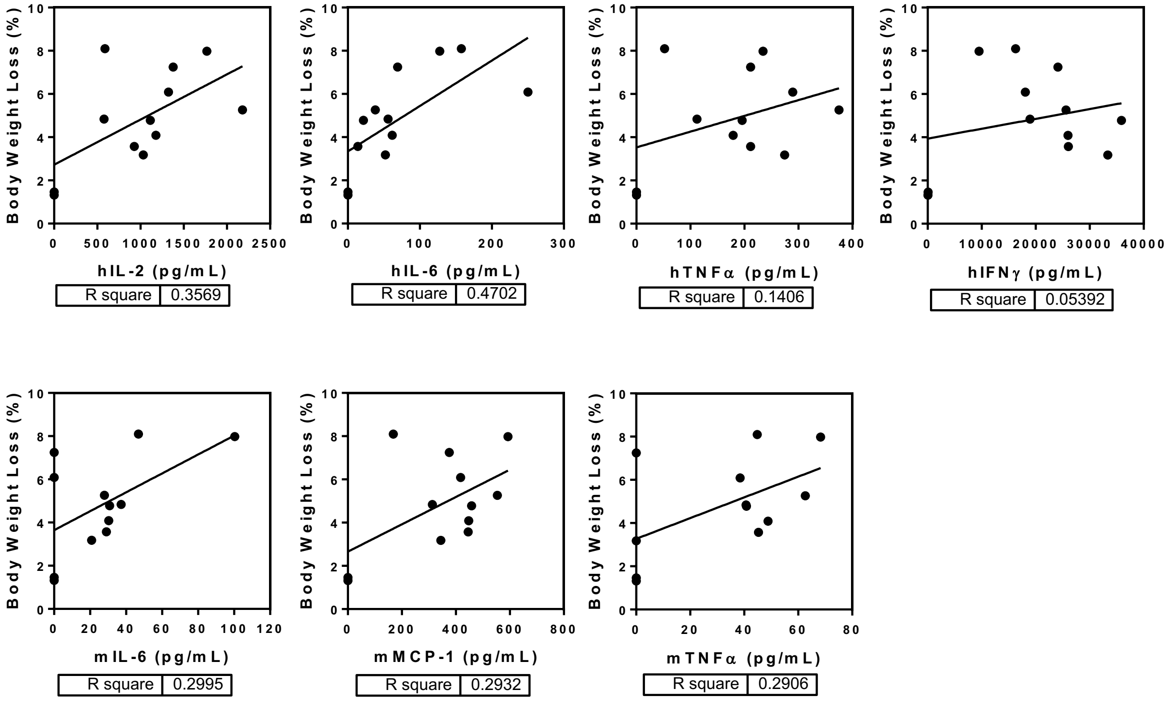

Supplemental Figure 1. Correlation between body weight loss and blood cytokine levels upon induction of CAR T cell therapy. NSG mice bearing various sizes of MDA-MB-231 tumors (150-850 mm^3) were treated with 10 $x10^6$ anti-fluorescein CAR T cells on day 0 and then with 500 nmole/kg FITC-folate on day 2. Body weight change and plasma cytokine levels were measured 18 hours after FITC-folate administration.

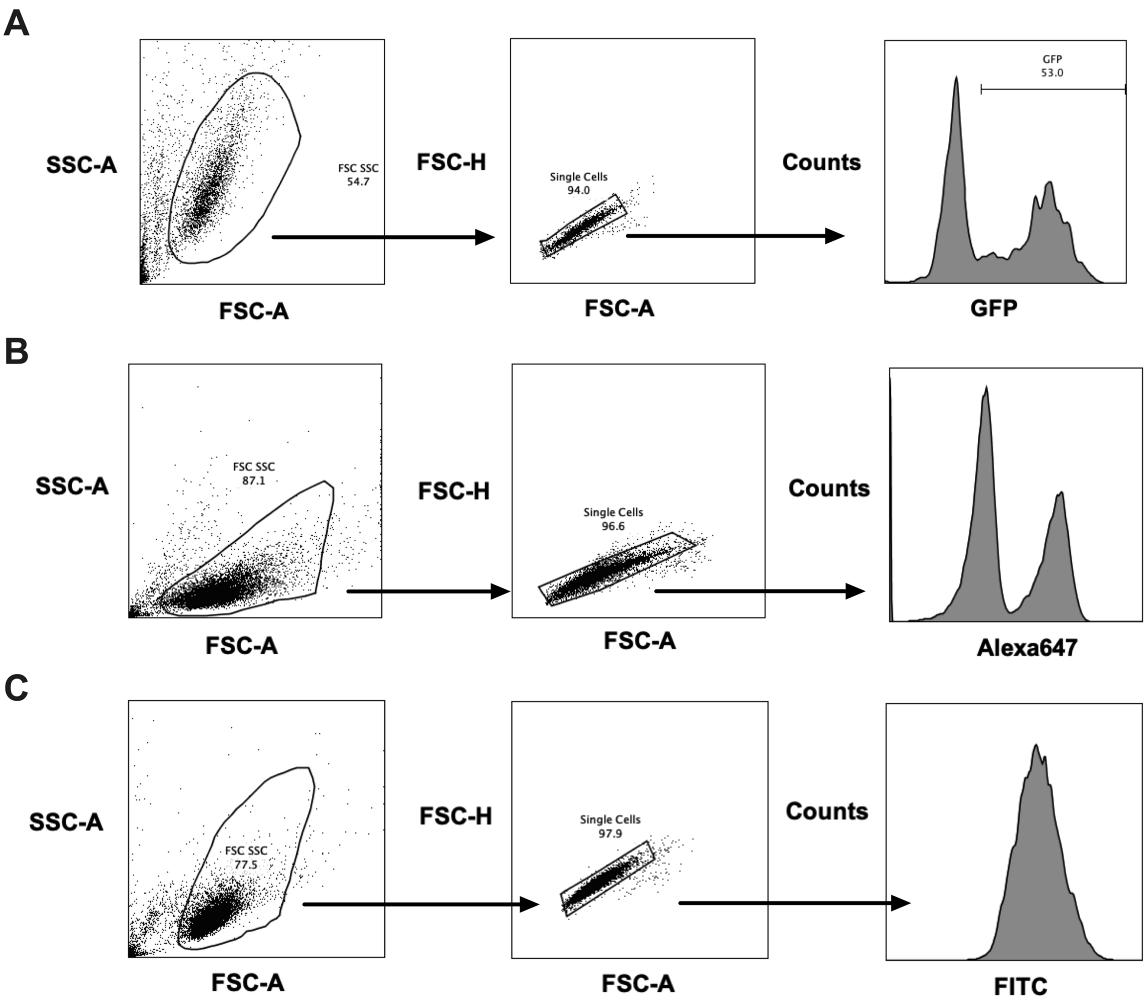

Supplemental Figure 2. Gating strategies used for flow cytometry analysis. (A) gating strategy to monitor CAR T cell transduction. (B) gating strategy to analysis binding of adapter to anti-fluorescein CAR on transduced T cells. (C) gating strategy to evaluate folate receptor expression on cancer cells.
